# Economic and Societal Impact of a Systems-of-Care Approach for STEMI Management in Low and Middle-Income Countries: Insights from the TN STEMI Program

**DOI:** 10.5334/aogh.2508

**Published:** 2019-10-15

**Authors:** Varshini Neethi Mohan, Thomas Alexander, V. R. Muraleedharan, Ajit Mullasari, Jagat Narula, Umesh N. Khot, Brahmajee K. Nallamothu, Dharam J. Kumbhani

**Affiliations:** 1 STEMI Care India, IN; 2 Department of Cardiology, Kovai Medical Center and Hospital, Coimbatore, Tamil Nadu, IN; 3 Department of humanities and social sciences, IIT Madras, Tamil Nadu, IN; 4 Department of Cardiology, Madras Medical Mission, Chennai, Tamil Nadu, IN; 5 Division of Cardiology, Mount Sinai Medical Center, New York, NY, US; 6 Heart and Vascular Institute, Cleveland Clinic, Cleveland, OH, US; 7 Department of Internal Medicine and Michigan Center for Health Analytics and Medical Prediction, University of Michigan, Ann Arbor, MI, US; 8 Department of Internal Medicine, Division of Cardiology, University of Texas Southwestern Medical Center, Dallas, TX, US

## Abstract

The TN STEMI Program was a multicenter, prospective, observational study conducted in Tamil Nadu, India, that assessed the effects of implementing the STEMI India Model for the management of STEMI. We discuss the economic and societal impact in this article. Given that the intervention resulted in an absolute mortality reduction of 3.4%, we calculated a number needed to treat of 30 patients. At an annualized project cost of INR 15.11 million, this approximately calculates to INR 193,749 (USD 3,311) per life saved. The utility of the TN-STEMI Program can be estimated to be 1,108 life-years. This calculates to approximately INR 13,643 (USD 233) per life-year saved. Our estimates will likely be of particular interest to policy makers in low and middle-income countries, where financial and resource constraints pose a perennial public health challenge.

Ischemic heart disease remains the leading cause of disability and premature death across the globe. Its burden is rising disproportionately among lower- and middle-income countries (LMICs) and populations; it is estimated that 80% of all cardiovascular deaths now occur in LMICs [1,2]. Further, it tends to impact younger working-age people in LMICs than in high-income countries, with tremendous direct and indirect economic consequences [3]. For instance, the cumulative economic loss from cardiovascular diseases in LMICs between 2011 and 2025 is projected to be approximately $3.76 trillion [4]. Among patients with CAD, STEMI remains a common, acute and frequently fatal manifestation, particularly if untreated. Compared with high-income countries (HICs), STEMI patients account for a greater proportion of ACS patients, and are typically younger with fewer comorbidities [5]. Sadly, a lot of the mortality and morbidity from STEMI could be prevented if well-established therapies were made readily and uniformly available. This is challenging in LMICs due to significant limitations in human and financial resources. Moreover, given the large population burdens of these countries, these gaps in care can be frequently invisible. Accordingly, it is imperative for LMICs to develop standardized systems of care for STEMI patients that are both clinically effective and cost-efficient.

A hub-and-spoke model (the STEMI India Model) was developed to attempt bridging these gaps.

## The TN STEMI Program

The TN STEMI Program was a multi-centre, prospective, observational study conducted in Tamil Nadu, India, that assessed the effects of implementing the STEMI India Model for the management of STEMI [6,7,8]. In this project, 2,420 patients with STEMI were evaluated in a pre (n = 898)/post (n = 1,522) implementation design. The study involved the creation of an integrated, regional quality improvement program that linked 35 “spoke” health centers to the four large PCI “hub” hospitals and leveraged recent developments in public health insurance schemes, emergency medical services, and health information technology to enhance patient access to care. This integrated system greatly improved process measures from pre- to post-implementation such as fibrinolysis to PCI time (39.2 vs. 17.3 hours, p = 0.003), performance of coronary angiography (35.0% vs. 60.8%, p < 0.0001) and primary PCI use (21.8% vs. 40.7%, p < 0.0001). The use of a pharmacoinvasive strategy also increased (13.1% vs. 20.1%, p < 0.0001), particularly among spoke hospital patients (2.5% vs. 28.2%, p < 0.0001). The implementation of this network was associated with significantly lower 1-year mortality (17.6% vs. 14.2%, aOR = 0.67; 95% CI 0.49–0.90; p = 0.01) [9].

## Economic considerations

Having established the feasibility and potential mortality benefit of implementing such a strategy, it is helpful to consider both the actuarial and estimated costs of implementing such a strategy in other settings. The implementation of the pilot project was funded by STEMI India, a non-governmental organisation, and by the Indian Council of Medical Research. Over a period of 319 days (10.6 months), the total implementation costs incurred (in 2013 INR) were Rs.13.2 million (annualized cost Rs. 15.11 million, or USD 258,268 at the annual average exchange rate in 2013) [10]. This is the direct cost purely of setting up, implementing and monitoring such a “hub and spoke” model and does not include drug, device or hospitalization costs. A breakdown of these costs is as below:

**Table:** 

COSTS	*Rs. mn*

**Setting up**	**7.34**
Devices	5.63
Software & Servers	1.70
**Implementing**	**5.79**
Telecom & IT	1.68
Operations	4.11
**Monitoring**	**1.98**
Logistics	0.39
Overheads	1.59
**TOTAL**	**15.11**

Given that the intervention resulted in an absolute mortality reduction of 3.4%, we calculated a number needed to treat (NNT) of 30 patients. Considered differently, if we annualize the enrolment of patients in the post-implementation arm, it would mean that implementing this protocol resulted in 78 fewer deaths. At an annualized project cost of INR 15.11 million, this roughly calculates to INR 193,749 (USD 3,311) per life saved.

## Societal perspective

We can similarly use data from this project to estimate the cost-utility (effectiveness) of such a program from a societal perspective. Typically, one would need to look at quality-adjusted life-years or similar utilities for assessing cost-effectiveness of such an intervention. This information is not directly available. However, as a crude indicator, we can estimate “life-years saved” based on the age at the instance of MI, and how long the patient would be expected to live based on prevalent life-expectancy rates, and assuming no other competing risks of death [11]. With this assumption, total life years saved for patients older than life-expectancy at time of index event is zero. The average life-expectancy in Tamil Nadu in 2010–14 was 72.7 years for women and 68.6 years for men [12]. This would thus result in 4,381 life-years lost pre-implementation and 3,273 life-years lost post; the utility of the TN STEMI Program can therefore be estimated to be 1,108 life-years. Assuming a uniform distribution of these over the 78 deaths averted approximates that the program adds 14 years to each of those patients’ lives. Again, considering an annualized expenditure of INR 15.11 million, this calculates to approximately INR 13,643 (USD 233) per life-year saved.

We can estimate the societal benefits even better from a productivity standpoint when we extrapolate this mortality difference in terms of net gain to the total economy due to the deaths averted. This can be considered as the sum of net present value of all future income of the patients at the instance of their index event [13]. Assuming that this value is greater pre-intervention, the difference between these values post-implementation and pre-implementation is the total economic benefit of the intervention itself. This perspective, in our opinion, is the most useful, as it accounts for all the potential costs and benefits, and can be extended with reasonable objectivity across countries irrespective of level of income. Accordingly, since we anticipate that this is the measure that will be most valuable to policy-makers, we present this in greater detail below to show the net impact of establishing systems of care for STEMI in this program.

The expected number of working years left for the patient at the instance j of heart attack T_j_ for men and for women is arrived at by adding, at each remaining year of the patient’s life (up to age 70, assuming that anyone older would not work), the probability that (s)he will both survive and be part of the labour force. The formula is as follows:

{T_j} = \mathop \sum \nolimits_{t = j}^{70} {s_{j,t}}\,\,. {l_t}

where:

- *s_j,t_* is the probability that a person of age j will survive to the end of age t
- *l_t_* is the labour force participation rate at age t

*s_j,t_* are matrices that are arrived at from the deaths per 1,000 for each age by gender [14]. Probability of death at age t is the cumulative deaths per 1,000 divided by 1,000 from age j up to age t. Probability of survival from age j to age t is the inverse (1 – probability of death at age t given one is already age j).

The data for labour force participation rate at age t *l_t_* is calculated from the overall labour force participation rate (LFPR) of Tamil Nadu.

LFPR\,\, = \,\,\,\frac{\begin{array}{l}\qquad no.\,\,of\,employed\,\, + \\no.\,\,of\,unemployed\,persons\end{array}}{{Total\,population}}\,\, \times \,\,1000

The LFPR (per 1000) for all persons of all ages according to usual status (principal and subsidiary) for Tamil Nadu is 454 [15]. The total number of men and women in the labour force (2,18,27,337 and 1,10,90,789 respectively) in Tamil Nadu is arrived at by multiplying the LFPR by the gender-wise population (3,61,37,975 and 3,60,09,055 according to the Census of India, 2011) [16]. Since age-wise distribution per 1,000 employed in Tamil Nadu is only available in 5-year ranges, we assumed uniform distribution per year within the 5-year range [15]. The population of Tamil Nadu by age was obtained from census data [16].

The total numbers of men and women at age t in the labour force *l_t_* are now calculated by multiplying the number employed per 1,000 at that age t by the total number employed, and the LFPR at each age is computed as the number employed divided by the population at that age.

The number of working years left T for men and women at age j is calculated as the summation of the product of probability of survival at a particular age and the WPR at that age for all ages from the present age until age 70 (as, with the assumptions above, T becomes 0 at age 70).

The present value of all expected future income for the number of working years left T is then calculated by adding up discounted labour income for T years as follows:

PV(I)\,\, = \,\,\sum\nolimits_{i\, = \,0}^T {I_0} \,\,{(1\,\, + \,\,g)^i}\,/\,\,{(1\,\, + \,\,r)^i}

where

- I_0_ is average labour income per capita in the present year
- T is the expected number of working years for the average person in a particular age group
- g is the annual rate of income growth
- r is the social discount rate

In keeping with recent guidance by the World Bank, for low- and middle-income countries [13].

- the discount rate (r) is set at 6%
- and the annual growth rate for real income per capita (g) is set at 3%

The average labour income per capita I is calculated by dividing the labour share of GDP by the total number of employed workers:

I\,\, = \,\,(GDP\,\cdot\,s)\,/\,w

where

- s is labour share of GDP
- w is total number of employed workers

The average GDP of India at current prices over the five years from 2010 to 2014, chosen as the study was conducted between 2011 and 2013, was INR 83,222 billion (USD 1,422.25 billion at the annual average exchange rate in 2013) [17]. The total number of workers or the total labour force *w* is also averaged for the same period of five years, and is 481,832,540. The labour share of GDP of India *s* is 0.496 [18]. The average labour income calculated using the formula is INR 85,662.37 (USD 1,464). The gender pay gap is not taken into consideration here.

The present value (PV) of income of all patients who died until the 1-year follow-up was calculated post- and pre-implementation. The decrease in the PV of lost income (income that did not happen because of death) from the pre-implementation period to the post-implementation period is the gain to the economy provided by the intervention. Annualising these numbers for 2,265 patients results in INR 228.7 million (USD 3.91 million) lost pre-implementation and INR 175.6 million (USD 3 million) lost post-implementation, which is INR 53.1 million (USD 908,000) gained in the economy due to the protocol.

## Discussion

A benefit of INR 53.1 million on an annualized project expenditure of INR 15.11 million translates to INR 3.52 gained for every rupee spent. A sensitivity analysis changing discount rates and growth rates in our calculations of the net present values of future incomes is set down in the table below:

**Table:** 

	Discount Rate				

		4%	6%	8%	10%

Growth Rate	3%	3.8	3.5	3.3	3.1
	6%	4.1	3.9	3.6	3.4
	8%	4.4	4.1	3.9	3.7
	10%	4.6	4.4	4.1	3.9
	12.0%	4.7	4.6	4.4	4.1

Given that the 10-year Indian government bond yield, a substitute for risk-free discount rate, was 6.6% at the time of writing and the baseline GDP growth rate for 2017–18 was 5.9%, a realistic cost benefit ratio is 3.98 [19].

As discussed above, these estimates will likely be of particular interest to policy-makers in LMICs, where financial and resource constraints pose a perennial public health challenge. From a global viewpoint, the WHO considers interventions to be cost-effective if they have ICERs that are less than three times gross national income (GNI) per capita [20]. In 2016, India’s GNI per capita was $1,670, or $6,490 after adjustment for purchasing power parity [21]. Our estimates above (USD 3,311 per life saved for implementing a dedicated network) are likely to be well within these thresholds, even after adding in costs of drug, device, hospitalization and medication, leading us to the conclusion that investments in STEMI care yield economic benefits to society that greatly exceed the incremental costs involved.

## Limitations

Our calculations and the discussion above are not framed around the traditional form of cost-effectiveness measures such as incremental cost-effectiveness ratio (ICER) given that granular patient-level cost details were not available. However, our endeavour is to provide an estimate of the modular cost of implementing a program such as the TN STEMI Program *de novo* in settings such as LMICs where such infrastructure is either non-existent or rudimentary. We have used fairly conservative estimates in our imputations; the actual benefits could be higher, but will vary based on country and region. These costs will vary with nature of the health care and social system to which the program is being added. It is reasonable to assume that, depending on the existing health care infrastructure and management, program costs would be substantially different. In contexts with better infrastructure and fewer managerial inefficiencies, costs could be lower; on the other hand, in contexts with worse infrastructure and less management structure to build on, costs could be much higher. At the same time, it is possible that the benefits could also be lower and higher in these two different contexts. However, other formal ICER analyses of implementing STEMI networks in various countries have yielded similar values. For instance, the ICER of utilizing primary PCI for all-comers with STEMI in China was estimated at USD 10,700 [22]. Similarly, the Catalan STEMI study from Spain estimated the ICER of implementing a STEMI-network to be Euro 4,355 (USD 5,383) [23]. We also only considered mortality, but further benefits can be expected from reductions in morbidity as well.

## Final thoughts

For LMICs, the sharp increase in mortality and morbidity from non-communicable diseases such as ischemic heart disease presents one of the biggest challenges to sustained economic growth in the 21^st^ century and beyond. Tragically, this is occurring despite tremendous strides in available management options for these conditions. For STEMI in particular, the current unstructured and highly inefficient system of management in most LMICs means that the gains from revascularization therapies noted in developed countries can seldom be achieved. Such a system puts the onus on patients (and their families), who are already in the throes of an MI, to have the wherewithal to present to the right hospital within the right timeframe and with the availability of immediate financial liquidity. We therefore believe that the large-scale adoption of a STEMI systems-of-care approach as studied in the TN STEMI Program represents a cost-effective investment for health systems to correct many such inequalities and social deprivation among this high-risk vulnerable patient population. Such investments will likely be repaid many times over in hundreds of thousands of lives saved each year, enhanced economic development, and strengthened global security.

## Additional File

The additional file for this article can be found as follows:

10.5334/aogh.2508.s1 Calculating T(j) by age j. Calculation of expected number of working years left T(j) at age j by gender using survival rate and labor force participation rate.
