# Supplementary material for: Economic and Societal Impact of a Systems-of-Care Approach for STEMI Management in Low and Middle-Income Countries: Insights from the TN STEMI Program
Source: Ann Glob Health. 2019 Oct 15;85(1):122. doi: 10.5334/aogh.2508 (PMC6798779; doi:10.5334/aogh.2508)
Supplement: Calculating T(j) by age j. — Calculation of expected number of working years left T(j) at age j by gender using survival rate and labor force participation rate. [file agh-85-1-2508-s1.pdf]

Supplemental table: calculating T(j) by age j

Calculation of expected number of working years left T(j) at age j by gender using survival rate and labour force participation rate

j	Age-wise distribution per 1000, male	Age-wise distribution (considering uniform dist. in 5-year range) per 1000, male	Tamil Nadu population by age, male	Age-wise number employed in TN, male	l(t), male	Age-wise distribution per 1000, female	Age-wise distribution (considering uniform dist. in 5-year range) per 1000, female	Tamil Nadu population by age, female	Age-wise number employed in TN, female	l(t), female	T(j) rounded off, male	T(j) rounded off, female
15	90	18	6,64,183	3,92,892	0.59	83	16.6	6,15,611	1,84,107	0.30	32	18
16		18	6,10,930	3,92,892	0.64		16.6	5,66,844	1,84,107	0.32	31	18
17		18	6,23,711	3,92,892	0.63		16.6	5,72,969	1,84,107	0.32	31	18
18		18	6,83,260	3,92,892	0.58		16.6	6,42,072	1,84,107	0.29	30	17
19		18	6,47,754	3,92,892	0.61		16.6	6,26,458	1,84,107	0.29	29	17
20	72	14.4	7,19,304	3,14,314	0.44	88	17.6	7,30,102	1,95,198	0.27	29	17
21		14.4	6,17,853	3,14,314	0.51		17.6	6,26,307	1,95,198	0.31	29	17
22		14.4	6,08,972	3,14,314	0.52		17.6	6,33,913	1,95,198	0.31	28	16
23		14.4	6,03,747	3,14,314	0.52		17.6	6,26,367	1,95,198	0.31	28	16
24		14.4	6,04,928	3,14,314	0.52		17.6	6,40,050	1,95,198	0.30	27	16
25	85	17	7,25,651	3,71,065	0.51	92	18.4	8,23,418	2,04,071	0.25	27	15
26		17	6,16,761	3,71,065	0.60		18.4	6,54,989	2,04,071	0.31	26	15
27		17	6,62,270	3,71,065	0.56		18.4	6,71,723	2,04,071	0.30	26	15
28		17	6,36,844	3,71,065	0.58		18.4	6,84,622	2,04,071	0.30	25	15
29		17	5,95,848	3,71,065	0.62		18.4	5,91,620	2,04,071	0.34	25	14
30	83	16.6	8,20,748	3,62,334	0.44	78	15.6	8,88,461	1,73,016	0.19	24	14
31		16.6	5,43,290	3,62,334	0.67		15.6	5,09,929	1,73,016	0.34	24	14
32		16.6	5,61,252	3,62,334	0.65		15.6	5,49,118	1,73,016	0.32	23	13
33		16.6	4,79,816	3,62,334	0.76		15.6	4,60,479	1,73,016	0.38	23	13
34		16.6	5,03,643	3,62,334	0.72		15.6	4,98,824	1,73,016	0.35	22	13
35	80	16	7,93,584	3,49,237	0.44	94	18.8	8,98,975	2,08,507	0.23	21	12
36		16	5,22,689	3,49,237	0.67		18.8	5,53,105	2,08,507	0.38	21	12
37		16	4,98,491	3,49,237	0.70		18.8	5,12,661	2,08,507	0.41	20	12
38		16	5,30,792	3,49,237	0.66		18.8	5,81,130	2,08,507	0.36	20	12
39		16	4,69,649	3,49,237	0.74		18.8	4,75,380	2,08,507	0.44	19	11
40	76	15.2	8,34,994	3,31,776	0.40	69	13.8	9,07,757	1,53,053	0.17	18	11
41		15.2	4,61,677	3,31,776	0.72		13.8	4,28,255	1,53,053	0.36	18	11
42		15.2	4,82,183	3,31,776	0.69		13.8	4,46,027	1,53,053	0.34	18	10
43		15.2	3,81,996	3,31,776	0.87		13.8	3,58,589	1,53,053	0.43	17	10
44		15.2	3,88,996	3,31,776	0.85		13.8	3,73,735	1,53,053	0.41	16	10
45	72	14.4	7,87,942	3,14,314	0.40	66	13.2	8,40,095	1,46,398	0.17	15	9
46		14.4	4,29,555	3,14,314	0.73		13.2	4,06,599	1,46,398	0.36	15	9
47		14.4	3,90,093	3,14,314	0.81		13.2	3,58,345	1,46,398	0.41	15	9
48		14.4	4,14,078	3,14,314	0.76		13.2	4,01,454	1,46,398	0.36	14	8
49		14.4	3,53,247	3,14,314	0.89		13.2	3,31,648	1,46,398	0.44	13	8
50	49	9.8	6,87,779	2,13,908	0.31	50	10	7,70,315	1,10,908	0.14	12	8
51		9.8	3,30,357	2,13,908	0.65		10	2,93,922	1,10,908	0.38	12	8
52		9.8	3,20,908	2,13,908	0.67		10	2,77,243	1,10,908	0.40	12	7
53		9.8	2,52,024	2,13,908	0.85		10	2,16,943	1,10,908	0.51	11	7
54		9.8	2,84,495	2,13,908	0.75		10	2,69,474	1,10,908	0.41	11	6
55	43	8.6	5,42,372	1,87,715	0.35	45	9	6,34,927	99,817	0.16	10	6
56		8.6	2,90,232	1,87,715	0.65		9	2,72,943	99,817	0.37	10	6
57		8.6	2,26,492	1,87,715	0.83		9	1,98,579	99,817	0.50	9	6
58		8.6	2,47,193	1,87,715	0.76		9	2,31,998	99,817	0.43	9	5
59		8.6	2,34,635	1,87,715	0.80		9	2,21,771	99,817	0.45	8	5
60	41	8.2	5,80,996	1,78,984	0.31	43	8.6	6,95,393	95,381	0.14	7	4
61		8.2	2,36,407	1,78,984	0.76		8.6	2,24,112	95,381	0.43	7	4
62		8.2	2,08,590	1,78,984	0.86		8.6	1,78,419	95,381	0.53	7	4
63		8.2	1,80,540	1,78,984	0.99		8.6	1,44,997	95,381	0.66	6	4
64		8.2	1,71,506	1,78,984	1.04		8.6	1,61,648	95,381	0.59	5	3
65	63	6.2	3,90,162	1,35,329	0.35	62	6.2	4,87,387	68,763	0.14	4	2
66		6.2	1,50,464	1,35,329	0.90		6.2	1,54,890	68,763	0.44	4	2
67		6.2	1,22,714	1,35,329	1.10		6.2	1,02,082	68,763	0.67	3	2
68		6.2	1,15,697	1,35,329	1.17		6.2	1,10,804	68,763	0.62	3	1
69		6.2	1,17,375	1,35,329	1.15		6.2	1,16,795	68,763	0.59	1	1
70		6.2	3,51,563	1,35,329	0.38		6.2	4,16,655	68,763	0.17	-	-
Sources:	NSS 68th Round, Key Indicators of Employment and Unemployment in India, 2011-12 (page 34), Tamil Nadu, male)		Age Data, Census of India (Tamil Nadu, row 3423, all persons)	Extrapolated from LFPR of Tamil Nadu		NSS 68th Round, Key Indicators of Employment and Unemployment in India, 2011-12 (page 35), Tamil Nadu, female)		Age Data, Census of India (Tamil Nadu, row 3423, all persons)	Extrapolated from LFPR of Tamil Nadu			
